# Effect of continuous dialysis on blood pH in acidemic hypercapnic animals with severe acute kidney injury: a randomized experimental study comparing high vs. low bicarbonate affluent

**DOI:** 10.1186/s40635-017-0141-6

**Published:** 2017-05-30

**Authors:** Thiago Gomes Romano, Luciano Cesar Pontes Azevedo, Pedro Vitale Mendes, Eduardo Leite Vieira Costa, Marcelo Park

**Affiliations:** 1 Nephrology Department, ABC Medical School, Av. Príncipe de Gales, 821, Príncipe de Gales, Santo André, São Paulo 09060-650 Brazil; 2 Research and Education Institute, Hospital Sírio-Libanês, São Paulo, Brazil; 3 Emergency Medicine Discipline, Hospital das Clínicas, University of São Paulo, São Paulo, Brazil; 4 Cardio-Pulmonary Department, Pulmonary Division, Heart Institute (Incor), University of São Paulo, São Paulo, Brazil

**Keywords:** Sodium bicarbonate, Dialysis solutions, Hypercapnia, Acute kidney injury, Respiratory insufficiency

## Abstract

**Background:**

Controlling blood pH during acute ventilatory failure and hypercapnia in individuals suffering from severe acute kidney injury (AKI) and undergoing continuous renal replacement therapy (CRRT) is of paramount importance in critical care settings. In this situation, the optimal concentration of sodium bicarbonate in the dialysate is still an unsolved question in critical care since high concentrations may worsen carbon dioxide levels and low concentrations may not be as effective in controlling pH.

**Methods:**

We performed a randomized, non-blinded, experimental study. AKI was induced in 12 female pigs via renal hilum ligation and hypoventilation by reducing the tidal volume during mechanical ventilation with the goal of achieving a pH between 7.10–7.15. After achieving the target pH, animals were randomized to undergo isovolemic hemodialysis with one of two bicarbonate concentrations in the dialysate (40 mEq/L [group 40] vs. 20 mEq/L [group 20]).

**Results:**

Hemodynamic, respiratory, and laboratory data were collected. The median pH value at CRRT initiation was 7.14 [7.12, 7.15] in group 20 and 7.13 [7.09, 7.14] in group 40 (*P* = ns). The median baseline PaCO_2_ was 74 [72, 81] mmHg in group 20 vs. 79 [63, 85] mmHg in group 40 (*P* = ns). After 3 h of CRRT, the pH value was 7.05 [6.95, 7.09] in group 20 and 7.12 [7.1, 7.14] in group 40 (*P* < 0.05), with corresponding values of PaCO_2_ of 85 [79, 88] mmHg vs. 81 [63, 100] mmHg (*P* = ns). The difference in pH after 3 h was due to a metabolic component [standard base excess −10.4 [−12.5, −9.5] mEq/L in group 20 vs. –7.6 [−9.2, −5.1] mEq/L in group 40) (*P* < 0.05)]. Despite the increased infusion of bicarbonate in group 40, the blood CO_2_ content did not change during the experiment. The 12-h survival rate was higher in group 40 (67% vs. 0, *P* = 0.032).

**Conclusions:**

A higher bicarbonate concentration in the dialysate of animals undergoing hypercapnic respiratory failure was associated with improved blood pH control without increasing the PaCO_2_ levels.

**Electronic supplementary material:**

The online version of this article (doi:10.1186/s40635-017-0141-6) contains supplementary material, which is available to authorized users.

## Background

As many as 25% of patients admitted to intensive care units require mechanical ventilation due to acute respiratory failure [1]. Acute kidney injury (AKI) is common in this scenario [2–4], resulting in nearly 20% of patients with hypercapnia and hypoxemic respiratory failure undergoing renal replacement therapy (RRT) [1].

Achieving an acceptable pH (≥7.20) through either bicarbonate infusion or RRT should mimic the physiological metabolic adaptation to respiratory acidemia [5, 6]. In chronically hypercapnic patients, renal adaptation to hypercapnia relies on elevating plasma bicarbonate and reducing serum chloride [7].

Notwithstanding, bicarbonate supplementation in patients with limited alveolar ventilation is a matter of controversy. Over 80% of the carbon dioxide (CO_2_) in blood exists in the form of bicarbonate, which is in equilibrium with other forms of CO_2,_ including the dissolved portion that can be readily eliminated by the lungs [8]. Sodium bicarbonate infusions shift this equilibrium and generate more dissolved CO_2_. Indeed, sodium bicarbonate infusions have been associated with elevations of the partial pressure of arterial CO_2_ (PaCO_2_) [9] and deterioration of clinical status [10, 11].

Conversely, while CRRT with low-bicarbonate replacement fluids can avoid CO_2_ retention in patients with limited alveolar ventilation, it is less adequate at correcting acidemia. Therefore, the aim of this study was to investigate the effect of higher (40 mEq/L) and lower (20 mEq/L) dialysate concentrations of bicarbonate on blood pH in acidemic hypercapnic animals with severe AKI, which would address an unsolved question in critical care.

## Methods

The swine Agroceres® from the breeder Minipig Pesquisa e Desenvolvimento (São Paulo, Brazil) with a median weight of 31 kg (range of 26–38 kg) was used. Animal care was conducted in accordance with institutional guidelines, and approval was obtained from the Animal Research Ethical Committee at the Teaching and Research Institute of Hospital Sírio Libanês, São Paulo, Brazil, under the protocol CEUA P 2013.09 (July 2013). The timeline of the experiment is shown in Additional file 1: Figure S1.

### Anesthesia and instrumentation of animals

Anesthesia pre-induction was performed with intramuscular ketamine 5.0 mg/kg and midazolam 0.5 mg/kg. Anesthesia was induced using intravenous boluses of propofol 8 mg/kg and remifentanil 6 μg/kg and maintained during the entire experiment with starting doses of remifentanil at 6–12 μg/kg/h, midazolam 0.5–1.0 mg/kg/h, and propofol 2–5 mg/kg/h. Normal saline infusion at 20 ml/kg/h was maintained until the stabilization period. Additional normal saline boluses of 250 ml were administered if either the mean arterial pressure (MAP) decreased below 65 mmHg or the heart rate (HR) increased above 110 beats/min despite analgosedation.

Animals were connected to an Evita XL® mechanical ventilator (Dräger™, Lübeck, Germany) and ventilated with 8–10 ml/kg of tidal volume (V_T_), a positive end-expiratory pressure (PEEP) of 5 cm H_2_O, inspired fraction of oxygen (FiO_2_) to achieve a peripheral oxygen saturation between 94 and 96%, and a respiratory rate titrated to an end-tidal carbon dioxide pressure (ETCO_2_) between 30 and 35 mmHg. Airway pressures, V_T_, and ETCO_2_ were monitored through a NICO® system (Dixtal Biomedica Ind Com™, SP, Brazil). Electrocardiography, heart rate, oxygen saturation, and systemic pressures were monitored with a multiparametric monitor (Infinity Delta XL®, Dräger™, Lübeck, Germany).

A central line was placed in the left femoral vein for infusions, an arterial line was placed in the right femoral artery for blood sampling collection and invasive pressure measurement, a dialysis catheter (12-French, 16 cm, Arrow™, PA, USA) was placed in the right jugular vein, and a pulmonary artery catheter (Edwards Lifesciences™, Irvine, USA) was placed in the left jugular vein. The pulmonary artery pressures were monitored with a multiparametric monitor, and cardiac output was continuously measured with a Vigilance II® monitor (Edwards Lifesciences™, Irvine, USA). Via median laparotomy, we ligated the renal veins, arteries, and ureters, and a cystostomy tube was surgically placed. Analgosedation was continuously monitored via heart rate, spontaneous movements, and blood pressure. Animals were euthanized with a bolus infusion of 10 ml of potassium chloride 19.1% at the end of the experiment.

### Stabilization period

After the instrumentation phase, animals underwent stabilization. The room temperature was set to 18 °C, and a Bair Hugger® external heating air blanket (3 M™, MN, USA) and Termopet® heating mattress (Styllus term™, São Paulo, Brazil) were adjusted to maintain the animal’s central temperature at 37–38 °C.

### Hypercapnia induction

The tidal volume was reduced to 2/3 of the initial settings. After 1 h and every 15 min thereafter, arterial blood samples were drawn. If necessary, V_T_ was adjusted by 0.5 ml/kg of body weight, aiming at a blood pH range of 7.10–7.15. Hypercapnia was considered stable if three consecutive arterial blood samples had a pH in the desired range and if the PaCO_2_ varied less than 3% in those measurements. After achieving stable hypercapnic conditions, baseline data were collected. After initiation of hypercapnia, if the MAP dropped below 65 mmHg, normal saline boluses were allowed up to 100 ml/kg, and persistent hypotension was managed with an infusion of norepinephrine.

### Randomization of animals

Animals were randomly allocated using a closed box containing sealed envelopes indicating one of the two groups just before baseline data collection. The two groups consisted of a low-bicarbonate group (20 mEq/L, group 20) and a high-bicarbonate group (40 mEq/L, group 40). We arbitrarily planned an initial cohort of 18 animals. Initially, 12 animals (6:6 scheme) were randomized, after which an additional 6 animals (3:3 scheme) would be added if necessary.

### Renal replacement therapy

Blood was pumped using a peristaltic portable roller pump with a flow controller (BsMed™, Guangzhou, China) at a rate of 6 ml/kg/min^−^, and the effluent and affluent were regulated by infusion pumps (Hospira™, II, USA) at a rate of 60 ml/kg/h in an isovolemic setting.

Affluent fluid control was checked by conducting infusion pump volume measurements and assessing the affluent weight loss using an available scale (Toledo do Brasil Ind Bal Ltda™, SP, Brazil). Compositions of the two dialysate solutions are shown in Additional file 2: Table S1. The filter used was a Fresenius F8® (Fresenius Medical Care™, MA, USA), a low-flow polysulphone filter with a *K*_uf_ of 7.5 ml h^−1^ mmHg^−1^ and a surface area of 1.8 m^2^. The pressures in the extracorporeal system were continuously monitored in the arterial (P1), pre-filter (P2), and in the venous lines (P3) using a Dixtal 2020® multiparametric monitor (Dixtal Biomedica Ind Com™, SP, Brazil).

Anticoagulation was achieved with systemic heparin administration with a bolus of 80 IU/kg and maintenance at 20 IU kg^−1^ h^−1^. The doses of heparin were checked every 6 h and adapted to achieve an activated coagulation time with a target of 2.0–2.5 times baseline. Calcium gluconate was infused at a rate of 0.5–1.0 mg kg^−1^ h^−1^ of elementary calcium. The RRT efficacy was evaluated by the ratio of fluid urea nitrogen to blood urea nitrogen (FUN/BUN, a value of 1 denotes the most efficient clearance) [12].

### Data collection and laboratory analysis

Venous and arterial blood samples were drawn for gas analysis and other laboratory tests (e.g., Na^+^, K^+^, Ca^2+^, Cl^−^, lactate, glucose, and hemoglobin). Data were collected after stabilization, during hypercapnia installation, and every hour until either death or 12 h after initiation of dialysis.

Venous blood samples for albumin, urea, creatinine, phosphate, and magnesium measurements were collected at the end of stabilization, upon hypercapnia installation, and 1, 3, 6, 9, and 12 h after dialysis initiation or until death. Dialysis effluent was sampled for the measurement of urea, creatinine, PCO_2_, and pH at 1, 3, 6, 9, and 12 h after dialysis initiation or until death.

Mg^2+^ was measured using a colorimetric technique, and phosphate was measured using an ultraviolet technique. Urea in both the blood and urine was measured with a kinetic technique, and albumin was measured with a bromocresol dye colorimetric technique. Blood gases, Na^+^, K^+^, Ca^2+^, Cl^−^, lactate, glucose, and hemoglobin, were analyzed on an OmniAnalyser (Roche Diagnostics System, F. Hoffmann-La Roche Ltd., Basel, Switzerland).

### Calculated variables

- Blood CO_2_ content (mL/min) [13] = (1−((0.0289 × Hb)/(3.352–0.456 × (Sat_b_O_2_/100) × (8.142, pH_b_)))) × 2.226 × 0.0307 + (0.00057 × (37, temperature)) + (0.00002 × (37, temperature)^2^) × P_b_CO_2_ × (1 + 10 ^(pHb–6.086)^ + (0.042 × (7.4, pH_b_)) + ((38, temperature) × 0.00472 + (0.00139 × (7.4, pH_b_))))
- Standard base excess (SBE–mEq/L) = 0.9287 × (HCO3^−^, 24.4 + 14.83 × (pH, 7.4)) [14]

### Statistical analysis

The main outcome was pH changes during the 12 h of dialysis treatment. Given the lack of published data on this outcome, a sample size calculation was not performed. Animals began to expire after 4 h of CRRT; therefore, data up to the 3rd hour after CRRT initiation (baseline, 1 and 3 h) were used for comparisons. At those timepoints, the albumin, phosphate, magnesium, urea, and creatinine measurements were all available. Another variable planned for consideration in the interim analysis was cumulative survival (log-rank test) during the 12-h experiment. To avoid the unnecessary use of animals, a target *P* value of <0.05 for pH difference was intended in all interim analyses.

Continuous data are presented as medians [interquartile range]. Continuous data over time for both groups were analyzed using interaction analyses with a fixed-effect of a mixed generalized model using the animals as a random factor. Post hoc analyses for interactions were performed using the Mann-Whitney test for inter-group analyses and the Wilcoxon tests for intra-group analyses. *P* < 0.05 was considered statistically significant. Kaplan-Meier cumulative survival curves and the log-rank test were used to evaluate the survival time from CRRT initiation to either death or 12 h of CRRT. Comparisons of the data acquired the final hour before death (in an effort to understand the causes of death) were performed using the Mann-Whitney test. R-Free statistical software (Vienna, Austria, 2009) was used for analysis and graph construction [14].

## Results

The study was stopped after the inclusion of the first 12 animals due to a statistically significant difference in the pH after 3 h of CRRT and in the survival between the groups. Hemodynamic, respiratory, and metabolic behavior of the animals during the PaCO_2_ equilibrium phase is shown in Additional file 3: Table S2. There were no differences between groups in the analyzed variables until the end of the PaCO_2_ equilibrium phase. After the induction of hypercapnia, we could notice that there was a drop in mean arterial pressure and pulmonary arterial pressure, and also, metabolic acidosis was induced along hypercapnic acidosis.

**Fig. 1:**
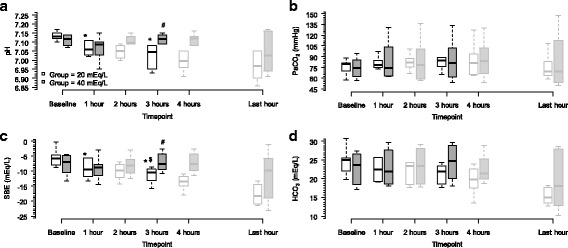
Temporal acid-base variation based on group. Panel **a** shows the pH changes over time (mixed model: group vs. pH interaction, *P* = 0.089; timepoint vs. pH interaction, *P* < 0.001; timepoint vs. group interaction, *P* = 0.003). Panel **b** shows the PaCO_2_ changes over time (mixed model: group vs. PaCO_2_ interaction, *P* = 0.913; timepoint vs. PaCO_2_ interaction, *P* = 0.311; timepoint vs. group interaction, *P* = 0.899). Panel **c** shows the SBE evolution over time (mixed model: group vs. SBE interaction, *P* = 0.02; timepoint vs. SBE interaction, *P* < 0.001, timepoint vs. group interaction, *P* < 0.001). Panel **d** shows the HCO_3_ changes over time (mixed model: group vs. HCO_3_ interaction, *P* = 0.401; timepoint vs. HCO_3_, *P* = 0.607; timepoint vs. group interaction, *P* = 0.664). SBE denotes standard base excess. Light gray boxplots were not used in analysis. *Wilcoxon post hoc analysis, *P* < 0.05 vs. baseline. #Mann-Whitney post hoc analysis, variable variation from baseline to 3 h; *P* < 0.05 vs. group 40. $Mann-Whitney post hoc analysis, *P* < 0.05 vs. group 40

**Fig. 2:**
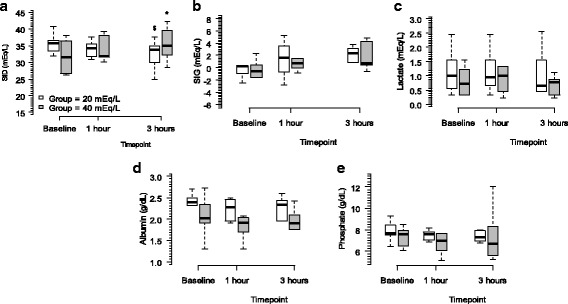
Temporal metabolic determinants of SBE variation based on group. Panel **a** shows the SID changes over time (mixed model: group vs. SIDa interaction, *P* = 0.021; timepoint vs. SIDa interaction, *P* < 0.001; timepoint vs. group interaction, *P* < 0.001). Panel **b** shows the SIG changes over time (mixed model: group vs. SIG interaction, *P* = 0.483; timepoint vs. SIG interaction, *P* = 0.797; timepoint vs. group interaction; *P* = 0.364). Panel **c** shows the lactate changes over the time (mixed model: group vs. lactate interaction, *P* = 0.473; timepoint vs. lactate interaction, *P* = 0.936; timepoint vs. group interaction, *P* = 0.871). Panel **d** shows the albumin changes over time (mixed model: group vs. albumin interaction, *P* = 0.182; timepoint vs. albumin interaction, *P* = 0.833; timepoint vs. group interaction, *P* = 0.432). Panel **e** shows the phosphate changes over time (mixed model: group vs. phosphate interaction, *P* = 0.565; timepoint vs. phosphate interaction, *P* = 0.450; timepoint vs. group interaction, *P* = 0.531). SID denotes strong ion difference. SIG denotes strong ion gap. SBE denotes standard base excess. *Wilcoxon post hoc analysis, *P* < 0.05 vs. baseline. $Mann-Whitney post hoc analysis, *P* < 0.05 vs. group 40

Figure 1 shows the pH, PaCO_2_, HCO_3_, and SBE values at baseline and after up to 12 h of CRRT. The pH upon initiating CRRT was 7.14 [7.12, 7.15] in group 20 and 7.13 [7.09, 7.14] in group 40 (*P* = ns). The pH value after 3 h of CRRT was 7.05 [6.95, 7.09] in group 20 and 7.12 [7.1, 7.14] in group 40 (*P* < 0.05). The PaCO_2_ was similar between the groups over time, and the variation in pH was due to the metabolic component as demonstrated by the elevated SBE in group 40 after 3 h of CRRT (−10.4 [−12.5, −9.5] mEq/L in group 20 vs. −7.6 [−9.2] in group 40) (*P* < 0.05). In Fig. 2 and Table 1, the metabolic components of the acid-base metabolism are described in detail, we could notice that SID was the main component of the acid-base metabolism that was different between both groups, and in Table 1, this difference in SID could be due to chloride levels which was significant higher in group 20. Additional file 4: Table S3 and Additional file 5: Table S4 show the hemodynamic and respiratory behavior of both groups from baseline to the last timepoint evaluated. The dose of norepinephrine was the only variable significantly different between groups, and higher dosage was needed in group 20, in order to keep hemodynamic targets described in the “Methods” section.

**Table 1:** Metabolic variables categorized according to the groups throughout the study

Variable	Group	Baseline ^a^	1 h^a^	2 h	3 h^a^	4 h	Last hour	*P* value
Sodium (mEq/L)	20 mEq/L	133 [131,137]	135 [132,138]	135 [134,141]	133 [138,136]	132 [130,134]	129 [128,131]	0.659^b^
	40 mEq/L	136 [133,138]	137 [135,139]	136 [133,137]	136 [132,136]	134 [132,136]	131 [119,131]	0.367^c^
Chloride (mEq/L)	20 mEq/L	105 [96,107]	107 [102,109]	108 [105,113]	107 [105,108]^f^	107 [105,110]	106 [105,109]	0.003^b^
	40 mEq/L	108 [106,113]	108 [105,112]	105 [103,113]	104 [101,111] ^d^	102 [101,112]	101 [95,112]	0.001^c^
Potassium (mEq/L)	20 mEq/L	4.4 [3.9,5.3]	5.0 [4,5.6]	4.9 [4.4,5.7]	5.9 [5.3,6.6]	6.4 [5.8,7.4]	6.6 [6.2,7.5]	0.079^b^
	40 mEq/L	4.4 [3.8, 4.8]	4.2 [3.7,5.3]	4.8 [4.3,6.0]	5.5 [4.6,6.4]	5.2 [4.6,6.5]	5.3 [4.9,7.0]	0.406^c^
Calcium (mEq/L)	20 mEq/L	1.22^e^ [1.12,1.35]	1.22 [0.86,1.27]	1.15 [0.99,1.35]	1.18 [1.15,1.21]^g^	1.14 [0.96,1.24]	1.02 [0.93,1.18]	0.226^b^
	40 mEq/L	1.42 [1.30,2.34]	1.27 [1.16,1.64]	1.17 [1.05,1.85]	1.17 [1.00,1.58]	2.26 [1.83,3.83]	1.07 [0.94,2.89]	0.021^c^
Magnesium (mEq/L)	20 mEq/L	2.55 [1.86,2.88	2.41 [1.85,3.37]	–	2.69 [2.20,3.52]	–	2.45 [2.02,3.35]	0.142^b^
	40 mEq/L	1.98 [1.53,3.45]	2.3 [1.47,3.8]	–	2.26 [1.83,3.83]	–	2.87 [2.09,4.0]	0.572^c^
Glucose (mg/dL)	20 mEq/L	101 [86,174]	111 [98,208]	87 [53,125]	102 [77,199]	100 [63,238]	132 [83,307]	0.892^b^
	40 mEq/L	114 [92,120]	90 [67,115]	106 [90,136]	103 [92,261]	98 [86,126]	81 [67,220]	0.351^c^
Temperature (°C)	20 mEq/L	38 [37.7,38.6]	37.0 [36.6,37.6]^d^	37.5 [36.8,37.9]	37.1 [36.7,37.7]^d^	37.5 [37.1,37.8]	36.5 [35.7,37.1]	0.011^b^
	40 mEq/L	37.9 [37.4,38.3]	37.7 [37.4,38]	37.7 [37.1,38.3]	37.7 [37.5,38.5]	37.3 [37.1,38.1]	37.5 [35.8,38.3]	0.445^c^
Hemoglobin (g/dL)	20 mEq/L	11.9 [10.9,12.6]	11.7 [10.4,13.0]	12.5 [8.8,13.4]	12.2 [9.8,13.7]	12.5 [9.3,14.4]	11.1 [7.15,12.2]	0.865^b^
	40 mEq/L	11.9 [11.2,13.8]	12.8 [11.2,14.4]	13.3 [12.3,14.0]	12.8 [11.6,13.8]	12.3 [11.5,14.9]	10.8 [8.5,13.7]	0.576^c^
Cumulative fluid balance (ml)	20 mEq/L	0 [0,0]	100 [0,225]	200 [150,375]	450 [238,625]^d^	450 [238,975]	700 [475,2150]	0.002^b^
	40 mEq/L	0 [0,0]	150 [0,275]	325 [75,425]	450 [275,600]^d^	650 [375,713]	1275 [425,1950]	0.654^c^

Data are shown as median [percentile 25th, percentile 75th]

^a^Only the timepoints baseline, 1st, 2nd, and 3rd hour were statistically analyzed

^b^Mixed model timepoint vs. variable interaction

^c^Mixed model group vs. variable interaction

^d^Wilcoxon post hoc analysis, *P* < 0.05 vs. baseline

^e^Mann-Whitney post hoc analysis, *P* < 0.05 vs. group 40

^f^Mixed model timepoint vs. group interaction *P* = 0.001. Mann-Whitney post hoc analysis chloride variation from baseline to 3 h *P* < 0.05 vs. group 40

^g^Mixed model timepoint vs. group interaction *P* = 0.046. Mann-Whitney post hoc calcium variation from baseline to 3 h *P* < 0.05 vs. group 40

**Fig. 3:**
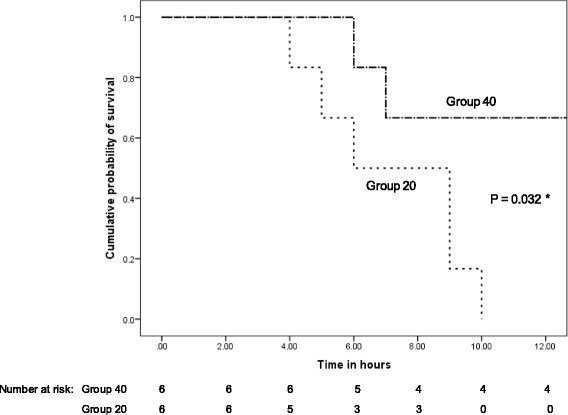
Kaplan-Meier survival analysis

The survival rate was clearly higher in group 40, and earliest death in group 20 occurred 4 h after initiating CRRT (Fig. 3). The median time of CRRT was 420 [225, 540] min in group 20 and 720 [450, 720] min in group 40 (*P* = 0.062). In the last hour before death, the pH was 6.97 [6.92, 7.03] vs. 7.03 [6.95, 7.04] (*P* = 0.630), the SBE was −18.2 [−20.3, −15.3] vs. −9.7 [−17.5, −5.9] mEq/L (*P* = 0.240), and the PaCO_2_ was 69 [65, 80] vs. 69 [55, 105] mmHg (*P* = 0.818) in groups 20 vs. 40, respectively. Among the hemodynamic variables measured in the last hour, only the systemic arterial pressure was statistically lower in group 20 than in group 40 (Additional file 3: Table S2).

**Fig. 4:**
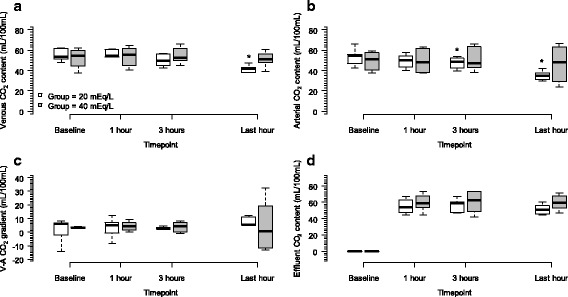
Temporal changes of CO_2_ content over RRT. Panel **a** shows the venous CO_2_ content evolution over the time (mixed model: group vs. venous CO_2_ content interaction, *P* = 0.230; timepoint vs. venous CO_2_ content interaction, *P* < 0.001; timepoint vs. group interaction, *P* < 0.001). Panel **b** shows the arterial CO_2_ content changes over time (mixed model: group vs. arterial CO_2_ content interaction, *P* = 0.231; timepoint vs. arterial CO_2_ content interaction, *P* < 0.001; timepoint vs. group interaction, *P* < 0.001). Panel **c** shows the venous-arterial CO_2_ gradient changes over time (mixed model: group vs. venous-arterial CO_2_ gradient interaction, *P* = 0.815; timepoint vs. venous-arterial CO_2_ gradient interaction, *P* = 0.919; timepoint vs. group interaction, *P* = 0.972). Panel **d** shows the effluent CO_2_ content changes over time (mixed model: group vs. effluent CO_2_ content interaction, *P* = 0.769; timepoint vs. effluent CO_2_ content interaction, *P* = 0.136; timepoint vs. group interaction, *P* = 0.242), for the effluent CO_2_ content, the baseline data were excluded. *Wilcoxon post hoc analysis, *P* < 0.05 vs. baseline

The CRRT efficacy variables recorded during the entire experiment are shown in Additional file 6: Table S5. CRRT efficacy variables were equals between groups, and the cumulative affluent and effluent volumes were obvious higher in group 40 due to longer CRRT duration in this arm. CO_2_ content in the blood from the arterial and venous lines, as well as the CO_2_ content in the effluent fluid, are shown in Fig. 4.

## Discussion

This study was designed to answer the clinical dilemma of bicarbonate concentrations in the dialysate of CRRT during hypercapnia by addressing which of two concentrations—40 or 20 mEq/L—resulted in better control of blood pH in hypercapnic animals with severe AKI. Our findings support that a higher bicarbonate dialysate concentration in acidemic hypercapnic animals has a better effect on pH control as early as 3 h after CRRT initiation as shown by a median reduction of 15% in the H^+^ concentration from a pH of 7.05 in group 20 to a pH of 7.12 in group 40; additionally, the increased concentration had a significant impact on mortality (100% in group 20 vs. 33% in group 40). Improved pH control was due to superior metabolic compensation secondary to the effects of CRRT, which was demonstrated by a higher SBE after 3 h. Interestingly, the total blood content of CO_2_ was not different between the groups, showing that respiratory component did not worsen despite the increased bicarbonate concentration in the dialysate.

Although the initial pH of our study seems to be extremely low, it is in accordance with the refractory academia definition used by Meade et al. and has been observed in 7% of patients with acute respiratory failure [15]. Carbon dioxide transport in the blood occurs in three forms as follows: dissolved in plasma (~5%), as bicarbonate (~80%), or bound to hemoglobin (~15%) [16]. Thus, one could argue that elevated bicarbonate concentrations in the dialysate can potentially worsen hypercapnia, especially in patients with limited alveolar ventilation [9, 17–19]. Conversely, removal of bicarbonate by CRRT with a low bicarbonate concentration in the dialysate (which has sieving and diffusing coefficients close to 1) could reduce total CO_2_ [20].

The blood pH of both groups decreased 1 h after initiating CRRT due to the metabolic content and was secondary to the increase in strong ion gap (SIG), which could be explained by the increase in unmeasured anions other than lactate such as pyruvate, sulphate, and citrate. In group 40, the pH returned to baseline levels 3 h after CRRT initiation, while in group 20, the pH continued to worsen. Worsening of acidemia in group 20 was mainly due to the metabolic component as indicated by the lower SBE and strong ion difference (SID) in this group. These findings support the use of CRRT with low-chloride and high-sodium bicarbonate solutions in hypercapnic animals to achieve better pH control.

Our findings could be well explained by the acid-base disturbance theory described by Peter Stewart [21], where pH and the H^+^ concentration are determined by the strong ion difference (SID), the total concentration of non-volatile weak acid (ATOT), and PCO_2_. The improved control of blood pH in the animals in group 40 was achieved by higher SID values, due to lower chloride infusion in this group.

Indeed, normally functioning kidneys exhibit similar results [7]. In healthy volunteers, CO_2_ retention triggers a metabolic response via renal sodium retention [22]. Rabbits exposed to high CO_2_ partial pressure (10%) for 52–56 h reabsorb bicarbonate through the Na^+^/HCO_3_^−^ co-transporter, resulting in increased serum bicarbonate and decreased serum chloride [23]. A link between hypochloremia and hypercapnia may manifest from the bicarbonate/chloride exchanger pendrin located in the apical domain of type B and non-A-non-B intercalated cells [24], and the expression of pendrin is down-regulated during hypercapnia, which provides collecting tubules the potential for increased bicarbonate reabsorption and chloride excretion [25]. Finally, these adaptations occur in acute as well as in chronic settings, with elevated urinary chloride excretion within 30 min after hypercapnia induction [7, 26, 27].

On average, the PaCO_2_ levels were similar between the groups. There are three non-mutually exclusive possible explanations for this observation. First, this result could be explained by Gattinoni’s “open system” theory in which CO_2_ clearance by the lungs attenuates the effect on blood pH of CO_2_ elevation secondary to sodium bicarbonate infusion; this theory contrasts the “closed system” theory in which the lungs are unable to clear CO_2_. In the latter situation, the lungs are unable to eliminate CO_2_ generated by the infusion of sodium bicarbonate; therefore, despite sodium infusion, an elevation on serum pH is not observed [28]. Acute respiratory distress syndrome, chronic obstructive pulmonary disease, and asthma are examples of “open systems” in which low-effective alveolar ventilation is associated with a preserved alveoli-capillary function [29, 30], and any increase in venous PaCO_2_ is associated with increased CO_2_ transfer in respiratory membranes [31]. In our study, we expected an increase in the total CO_2_ venous blood content in group 40 secondary to the influx of bicarbonate from dialysate, which would result in increased CO_2_ transfer in the lungs. However, neither the venous blood CO_2_ content nor the venous-arterial difference of CO_2_ was different between the groups; thus, it is difficult to ascribe the lack of PaCO_2_ elevation to enhanced pulmonary elimination. Second, CO_2_ has a large volume of distribution across different tissues and binding molecules, which might have blunted the observed effect in terms of PaCO_2_ (which is measured in the serum) [17, 32]. Finally, a slow infusion of bicarbonate can increase pH without significantly elevating PaCO_2_ [33]. Intermittent dialysis, which demands high dialysate flow, is known to be related to rising serum CO_2_ [10, 19]. On the other hand, a long-term CRRT with a high concentration of bicarbonate (32 mEq/L) has a non-significant effect on PaCO_2_ [34].

The survival was clearly different between the groups. All animals in group 20 died within the first 12 h of CRRT with a lower MAP despite the administration of high dosages of norepinephrine, nominally elevated pulmonary vascular resistance and reduced right ventricle systolic work (although these differences were not statistically significant). These findings strongly suggest that hemodynamic failure in group 20 was mainly associated with pulmonary-related right ventricle dysfunction, which could be secondary to more accentuated acidemia [35].

There were several potential limitations of our study. First, the sample size was small but in accordance with the animal research principles regarding the utilization of the lowest number of animals possible. Second, as an experimental study, the results cannot be directly extrapolated to the clinic; however, our results can serve as an important guide to building the rationale of CRRT during hypercapnia. Third, we did not assess the long-term effects of elevated PaCO_2_ levels on physiology and outcomes, which requires further extensive discussion [36]; this limitation would have been mitigated if we found a significant difference in PaCO_2_ between the groups. Fourth, we only studied healthy lungs, which, although they were severely hypoventilated, had preserved alveolocapillary membranes. Fifth, our model of kidney injury induction may cause an inflammatory response and hypercatabolism; however, this response is only found in acute and critically ill subjects. Finally, blinding was not possible because the investigators were responsible for preparing the dialysate solution.

## Conclusions

Dialysate with 40 mEq/L of sodium bicarbonate is more efficient than 20 mEq/L in controlling pH during acidemia in hypercapnic animals with severe AKI. The clinical concern that respiratory acidemia may worsen because of a high bicarbonate concentration was not supported in this experimental study.

## Additional files

Additional file 1: Figure S1. Timeline of the study. After the CRRT initiation, arterial blood gas analyses were collected every hour; however, the highlighted points in the figure were the analyzed timepoints. (DOCX 19 kb)

Additional file 2: Table S1. Dialysate composition of the two solutions used in the experiment (DOCX 14 kb)

Additional file 3: Table S2. Hemodynamics and respiratory and metabolic variables collected during the equilibrium phase. * Wilcoxon’s test for paired samples. (DOCX 18 kb)

Additional file 4: Table S3. Hemodynamic variables categorized throughout the study according to group. Data are shown as the median [25th percentile, 75th percentile]. § Only the timepoints at baseline and at 1, 2, and 3 h after CRRT initiation were statistically analyzed. *Mixed model timepoint vs. variable interaction. #Mixed model group vs. variable interaction. $Wilcoxon post hoc analysis, *P* < 0.05 vs. baseline. @ Mann-Whitney post hoc analysis, *P* < 0.05 vs. group 40. &Mixed model timepoint vs. group interaction, *P* = 0.001. Mann-Whitney post hoc analysis of chloride variations from baseline to 3 h, *P* < 0.05 vs. group 40. %Mixed model timepoint vs. group interaction, *P* = 0.046. Mann-Whitney post hoc analysis of calcium variations from baseline to 3 h, *P* < 0.05 vs. group 40. **Mann-Whitney, *P* < 0.05 vs. group 40. (DOCX 25 kb)

Additional file 5: Table S4. Respiratory variables categorized throughout the study based on the group. Data are shown as the median [25th percentile, 75th percentile]. § Only the data collected at baseline and at 1, 2, and 3 h after initiating CRRT were statistically analyzed. *Mixed model timepoint vs. variable interaction. #Mixed model group vs. variable interaction. (DOCX 21 kb)

Additional file 6: Table S5. Hemodialysis quality assessment. Data are shown as the median [25th percentile, 75th percentile]. *Mixed model timepoint vs. variable interaction. #Mixed model group vs. variable interaction. (DOCX 21 kb)

## Abbreviations

AKI: Acute kidney injury
BUN: Blood urea nitrogen
CO_2_: Carbon dioxide
CRRT: Continuous renal replacement therapy
ETCO_2_: End-tidal carbon dioxide pressure
FiO_2_: Fraction inspired of oxygen
FUN: Fluid urea nitrogen
HCO_3_^−^: Bicarbonate
MAP: Mean arterial pressure
Na^+^: Sodium
PEEP: Positive end-expiratory pressure
SBE: Standard base excess
V_T_: Tidal volume
