# Supplementary material for: Effect of continuous dialysis on blood pH in acidemic hypercapnic animals with severe acute kidney injury: a randomized experimental study comparing high vs. low bicarbonate affluent
Source: Intensive Care Med Exp. 2017 May 30;5:28. doi: 10.1186/s40635-017-0141-6 (PMC5449359; doi:10.1186/s40635-017-0141-6)
Supplement: Supplementary file 1 — Timeline of the study. After the CRRT initiation, arterial blood gas analyses were collected every hour; however, the highlighted points in the figure were the analyzed timepoints. (DOCX 19 kb) [file 40635_2017_141_MOESM1_ESM.docx]

**Figure S1:** Timeline of the study. After the CRRT initiation, arterial blood gas analyses were collected every hour; however the highlighted points in the figure were the analyzed time points.

CRRT denotes Continuous renal replacement therapy

* These are the points where laboratorial, metabolic, hemodynamic and respiratory data were collected.
