# Supplementary material for: Effect of continuous dialysis on blood pH in acidemic hypercapnic animals with severe acute kidney injury: a randomized experimental study comparing high vs. low bicarbonate affluent
Source: Intensive Care Med Exp. 2017 May 30;5:28. doi: 10.1186/s40635-017-0141-6 (PMC5449359; doi:10.1186/s40635-017-0141-6)
Supplement: Supplementary file 2 — Dialysate composition of the two solutions used in the experiment (DOCX 14 kb) [file 40635_2017_141_MOESM2_ESM.docx]

Table S1. Dialysate composition of the two solutions used in the experiment

| **20mEq/L of bicarbonate** | **40 mEq/L of bicarbonate** |
| --- | --- |
| - Na^+^ = 140 mEq / L - Cl^-^ = 120 mEq / L - HCO_3_^-^ = 20 mEq / L - MgSO_4_ = 2,0 mEq / L | - Na^+^ = 140 mEq / L - Cl^-^ = 100 mEq / L - HCO_3_^-^ = 40 mEq / L - MgSO_4_ = 2,0 mEq / L |
