# Supplementary material for: Effect of continuous dialysis on blood pH in acidemic hypercapnic animals with severe acute kidney injury: a randomized experimental study comparing high vs. low bicarbonate affluent
Source: Intensive Care Med Exp. 2017 May 30;5:28. doi: 10.1186/s40635-017-0141-6 (PMC5449359; doi:10.1186/s40635-017-0141-6)
Supplement: Supplementary file 3 — Hemodynamics and respiratory and metabolic variables collected during the equilibrium phase. * Wilcoxon’s test for paired samples. (DOCX 18 kb) [file 40635_2017_141_MOESM3_ESM.docx]

| Table S2: Hemodynamics, respiratory and metabolic variables during equilibrium phase | | | |
| --- | --- | --- | --- |
| Variable | Before hypercapnia | After hypercapnia | P value * |
|  |  |  |  |
| ***Hemodynamics*** |  |  |  |
| PAPm (mmHg) | 29 [26,34] | 34 [29,41] | 0.036 |
| PAm (mmHg) | 118 [98,128] | 92 [88,115] | 0.0006 |
| HR (beats per min) | 65 [51,84] | 85 [57,101] | 0.050 |
| CO (ml/min) | 2.0 [1.8,2.5] | 2.4 [1.9,2.7] | 0.239 |
| SvO_2_ (%) | 60 [40,67] | 64 [40,72] | 0.239 |
|  |  |  |  |
| ***Respiratory*** |  |  |  |
| Tidal volume (ml) | 278 [252,328] | 179 [146,202] | 0.002 |
| FiO_2_ (%) | 0.25 [0.25,0.38] | 0.33 [0.25,0.44] | 0.097 |
| Respiratory rate (breaths/min) | 30 [26,30] | 30 [26,30] | 0.345 |
| EtCO_2_ (mmHg) | 38 [34,41] | 93 [74,101] | 0.002 |
| SataO_2_ (%) | 100 [97,100] | 93 [90,96] | 0.012 |
| P/F ratio | 425 [380,465] | 313 [225,392] | 0.063 |
|  |  |  |  |
| ***Metabolic*** |  |  |  |
| pHa | 7.41 [7.32,7.49] | 7.13 [7.10,7.14] | 0.005 |
| PaCO_2_ (mmHg) | 31 [29,34] | 79 [63,85] | 0.002 |
| HCO_3_ (mEq/L) | 20 [16,24] | 25 [20,26] | 0.002 |
| SBE (mEq/L) | -2.4 [-8.8,0.1] | -6.1 [-8.8,-4.7] | 0.005 |
| SID (mEq/L) | 31 [29,34] | 34 [30,37] | 0.012 |
| SIG (mEq/L) | 1.1 [-2.2,3.4] | -0.5 [-2.4,0.3] | 0.063 |
| Lactate (mmol/L) | 1.4 [0.9,2.3] | 0.9 [0.4,1.7] | 0.025 |
| Na (mEq/L) | 137 [134,138] | 134 [133,137] | 0.062 |
| K (mEq/L) | 3.8 [3.4,4.2] | 4.4 [3.9,4.8] | 0.007 |
| Cai (mEq/L) | 1.11 [0.84,1.28] | 1.33 [1.19,1.45] | 0.009 |
| BUN (mg/dL) | 8 [ 5,12] | 8 [6,14] | 0.063 |
| Creatinine (mg/dL) | 1.6 [1.3,2.4] | 1.7 [1.5,2.3] | 0.254 |
| Mg (mEq/L) | 2.1 [1.7,3.4] | 2.6 [1.7,3.4] | 0.255 |
| Cl (mEq/L) | 110 [106,115] | 107 [105,110] | 0.036 |
| Alb (mg/dL) | 6.7 [5,7.7] | 5.6 [4.8,6.2] | 0.0004 |
| PO_4_ (mg/dL) | 6.5 [5.8,7.6] | 7.6 [6.8,8.5] | 0.063 |
| Temperature (Celsius) | 38.2 [37.7,38.3] | 37.9 [37.7,38.4] | 0.952 |
|  |  |  |  |

* Wilcoxon’s test for paired samples
