# Supplementary material for: Effect of continuous dialysis on blood pH in acidemic hypercapnic animals with severe acute kidney injury: a randomized experimental study comparing high vs. low bicarbonate affluent
Source: Intensive Care Med Exp. 2017 May 30;5:28. doi: 10.1186/s40635-017-0141-6 (PMC5449359; doi:10.1186/s40635-017-0141-6)
Supplement: Supplementary file 4 — Hemodynamic variables categorized throughout the study according to group. Data are shown as the median [25th percentile, 75th percentile]. § Only the timepoints at baseline and at 1, 2, and 3 h after CRRT initiation were statistically analyzed. *Mixed model timepoint vs. variable interaction. #Mixed model group vs. variable interaction. $Wilcoxon post hoc analysis, P < 0.05 vs. baseline. @ Mann-Whitney post hoc analysis, P < 0.05 vs. group 40. &Mixed model timepoint vs. group interaction, P = 0.001. Mann-Whitney post hoc analysis of chloride variations from baseline to 3 h, P < 0.05 vs. group 40. %Mixed model timepoint vs. group interaction, P = 0.046. Mann-Whitney post hoc analysis of calcium variations from baseline to 3 h, P < 0.05 vs. group 40. **Mann-Whitney, P < 0.05 vs. group 40. (DOCX 25 kb) [file 40635_2017_141_MOESM4_ESM.docx]

| **Table S3: Hemodynamic variables categorized according to the groups throughout the study** | | | | | | | | |
| --- | --- | --- | --- | --- | --- | --- | --- | --- |
|  |  |  |  |  |  |  |  |  |
| Variable | Group | Baseline ^§^ | 1 hour ^§^ | 2 hours | 3 hours ^§^ | 4hours | Last hour | P value |
|  |  |  |  |  |  |  |  |  |
| Heart Rate  (Beats/minute) | 20 mEq/L | 63  [55,119] | 115  [67,181] | 162 [83,187] | 162  [95,182] | 172 [144,191] | 137  [126,156] | 0.080 * |
|  | 40 mEq/L | 93  [74,107] | 152 [117,169] | 141 [108,174] | 137 [117,166] | 153 [115,186] | 144 [92,188] | 0.475 ^#^ |
|  |  |  |  |  |  |  |  |  |
| Mean ABP  (mmHg) | 20 mEq/L | 88  [79,100] | 75  [71,79] | 65  [57,80] | 71  [61,77] | 66  [47,81] | 31**  [26,34] | 0.253 * |
|  | 40 mEq/L | 98  [90,122] | 85  [70,96] | 84  [80,96] | 77  [68,90] | 70  [60,86] | 49  [43,71] | 0.151 ^#^ |
|  |  |  |  |  |  |  |  |  |
| Mean PAP  (mmHg) | 20 mEq/L | 37  [31,42] | 38  [34,43] | 38  [30,45] | 40  [29,50] | 36  [33,49] | 32  [30,35] | 0.513 * |
|  | 40 mEq/L | 31  [28,36] | 33  [30,41] | 34  [31,35] | 36  [34,38] | 36  [34,38] | 31  [27,33] | 0.625 ^#^ |
|  |  |  |  |  |  |  |  |  |
| CVP  (mmHg) | 20 mEq/L | 11  [6,14] | 11  [2,15] | 10  [5,15] | 9  [5,11] | 9  [6,13] | 9  [4,22] | 0.353 * |
|  | 40 mEq/L | 12  [7,14] | 11  [8,12] | 11  [9,12] | 11  [8,12] | 10  [6,11] | 9  [8,11] | 0.729 ^#^ |
|  |  |  |  |  |  |  |  |  |
| Pulmonary wedge pressure  (mmHg) | 20 mEq/L | 11  [3,15] | 10  [3,15] | 14  [9,23] | 13  [7,19] | 12  [3,17] | 8  [5,21] | 0.241 * |
|  | 40 mEq/L | 13  [10,15] | 12  [10,14] | 12  [9,16] | 14  [10,15] | 10  [8,15] | 10  [5,14] | 0.262 ^#^ |
|  |  |  |  |  |  |  |  |  |
| SvO_2_  (%) | 20 mEq/L | 52  [45,65] | 70  [58,78] | 64  [46,70] | 67  [51,69] | 58  [53,63] | 51  [20,67] | 0.270 * |
|  | 40 mEq/L | 67  [58,72] | 60  [50,70] | 63  [49,73] | 61  [44,74] | 60  [48,68] | 62  [30,84] | 0.221 ^#^ |
|  |  |  |  |  |  |  |  |  |
| Cardiac output  (ml/min) | 20 mEq/L | 2.15 [1.70,2.70] | 2.20 [1.82,2.50] | 1.85 [1.55,2.52] | 2.15 [1.72,2.75] | 2.30 [1.25,3.50] | 1.10  [0.90,3.90] | 0.315 * |
|  | 40 mEq/L | 2.50  [2.10,2.80] | 2.90 [1.75,3.22] | 3.00 [1.80,3.22] | 3.10  [2.60,8.10] | 2.70  [2.00,3.30] | 2.50 [1.20,3.73] | 0.484 ^#^ |
|  |  |  |  |  |  |  |  |  |
| Systemic vascular resistance | 20 mEq/L | 2962 [2427,3761] | 2358 [1928,2961] | 2517 [1828,3171] | 2237 [2001,2572] | 1804  [1424,2681] | 1056  [457,1843] | 0.98 * |
|  | 40 mEq/L | 2941 [2481,4308] | 2518 [1702,2931] | 2277 [1870,3211] | 1920 [910,2500] | 1971  [1616,2507] | 1558 [900,2131] | 0.404^#^ |
|  |  |  |  |  |  |  |  |  |
| Pulmonary vascular resistance | 20 mEq/L | 822 [659,1528] | 1066 [728,1572] | 776 [556,1264] | 928 [649,1414] | 965 [717,1560] | 1000 [490,2051] | 0.939* |
|  | 40 mEq/L | 652 [475,1008] | 717 [519,1018] | 592 [490,844] | 553 [363,785] | 710 [635,1153] | 584 [301,1355] | 0.241# |
|  |  |  |  |  |  |  |  |  |
| Left ventricule sysptolic work | 20 mEq/L | 32  [23,37] | 19  [12,24] | 11  [6,18] | 12  [6,25] | 10  [3,21] | 2  [1,7] | 0.105* |
|  | 40 mEq/L | 31  [27,38] | 19  [10,29] | 20  [10,35] | 23  [16,62] | 15  [9,25] | 12  [3,99] | 0.517^#^ |
|  |  |  |  |  |  |  |  |  |
| Right ventricule systolic work | 20 mEq/L | 11  [7,12] | 9  [5,12] | 6  [3,10] | 6  [3,14] | 5  [2,13] | 3  [2,5] | 0.236* |
|  | 40 mEq/L | 7  [5,9] | 6  [3,10] | 6  [3,10] | 6  [4,10] | 9  [6,22] | 7  [5,9] | 0.190^#^ |
|  |  |  |  |  |  |  |  |  |
| Animals on norepinephrine | 20 mEq/L | 0 | 1 | 3 | 4 | 5 | 5 |  |
|  | 40 mEq/L | 2 | 2 | 2 | 2 | 3 | 5 |  |
|  |  |  |  |  |  |  |  |  |
| Norepinephrine dosage (mcg/kg/min) | 20 mEq/L | 0  [0,0] | 0  [0,0] | 0  [0,0.4] | 0.3  [0,2.0] | 1.1  [0.1,5.0] | 5.5  [1.6,8.0] | 0.004* |
|  | 40 mEq/L | 0  [0,0.1] | 0  [0,0.4] | 0  [0,0.5] | 0  [0,0.7] | 0.3  [0,2.0] | 1.7 [0.8,10.0] | 0.21^#^ |

Data are shown as median [Percentile 25^th^,Percentile 75^th^].

§ Only the timepoints baseline, 1^st^, 2^nd^, and 3^rd^ hour were statistically analyzed.

* Mixed model timepoint vs. variable interaction.

# Mixed model group vs. variable interaction.

$ Wilcoxon *post-hoc* analysis, p < 0.05 vs. baseline.

@ Mann-Whitney *post-hoc* analysis, p < 0.05 vs. group 40.

& Mixed model timepoint vs. group interaction p = 0.001. Mann-Whitney *post-hoc* analysis chloride variation from baseline to 3 hours p < 0.05 vs. group 40.

% Mixed model timepoint vs. group interaction p = 0.046. Mann-Whitney *post-hoc* calcium variation from baseline to 3 hours p < 0.05 vs. group 40.

** Mann-Whitney, p < 0.05 vs group 40
