# Supplementary material for: Effect of continuous dialysis on blood pH in acidemic hypercapnic animals with severe acute kidney injury: a randomized experimental study comparing high vs. low bicarbonate affluent
Source: Intensive Care Med Exp. 2017 May 30;5:28. doi: 10.1186/s40635-017-0141-6 (PMC5449359; doi:10.1186/s40635-017-0141-6)
Supplement: Supplementary file 5 — Respiratory variables categorized throughout the study based on the group. Data are shown as the median [25th percentile, 75th percentile]. § Only the data collected at baseline and at 1, 2, and 3 h after initiating CRRT were statistically analyzed. *Mixed model timepoint vs. variable interaction. #Mixed model group vs. variable interaction. (DOCX 21 kb) [file 40635_2017_141_MOESM5_ESM.docx]

| **Table S4: Respiratory variables categorized to the groups throughout the study** | | | | | | | | |
| --- | --- | --- | --- | --- | --- | --- | --- | --- |
|  |  |  |  |  |  |  |  |  |
| Variable | Group | Baseline ^§^ | 1 hour ^§^ | 2 hours | 3 hours ^§^ | 4hours | Last hour | P value |
|  |  |  |  |  |  |  |  |  |
| Respiratory rate  (Breaths/minute) | 20 mEq/L | 29  [25,30] | 29  [25,30] | 29  [25,30] | 29  [25,30] | 29  [25,30] | 29  [25,30] | 0.990 * |
|  | 40 mEq/L | 30  [29,30] | 30  [29,30] | 30  [ 29,30] | 30  [ 29,30] | 30  [ 29,30] | 30  [ 29,30] | 1.000 ^#^ |
|  |  |  |  |  |  |  |  |  |
| Tidal volume  (mL) | 20 mEq/L | 183 [153,207] | 174 [151,198] | 173 [151,213] | 177 [152,219] | 172 [148,215] | 151 [100,222] | 0.523 * |
|  | 40 mEq/L | 166 [140,214] | 163 [140,208] | 166 [141,209] | 163 [142,207] | 163 [143,208] | 166 [146,210] | 1.000 ^#^ |
|  |  |  |  |  |  |  |  |  |
| FiO_2_ | 20 mEq/L | 0.37 [0.26,0.45] | 0.40 [0.34,0.49] | 0.40 [0.34,0.49] | 0.43 [0.39,0.53] | 0.43 [0.39,0.53] | 0.40 [0.38,0.48] | 0.736 * |
|  | 40 mEq/L | 0.30 [0.25,0.39] | 0.40 [0.36,0.43] | 0.40 [0.4,0.45] | 0.40 [0.40,0.43] | 0.40 [0.40,0.43] | 0.40 [0.40,0.43] | 0.457 ^#^ |
|  |  |  |  |  |  |  |  |  |
| Peak pressure (cmH_2_O) | 20 mEq/L | 21  [15,26] | 21  [16,25] | 20  [16,23] | 20  [17,22] | 21  [17,24] | 20  [16,23] | 0.944 * |
|  | 40 mEq/L | 25  [21,32] | 23  [20,25] | 23  [21,24] | 22  [20,25] | 23  [20,25] | 24  [21,29] | 0.146 ^#^ |
|  |  |  |  |  |  |  |  |  |
| Plateau pressure (cmH_2_O) | 20 mEq/L | 16  [11,18] | 14  [12,17] | 14  [12,16] | 14  [11,16] | 16  [13,19] | 15  [13,20] | 0.987 * |
|  | 40 mEq/L | 18  [12,23] | 15  [13,20] | 15  [13,17] | 13  [13,18] | 14  [14,18] | 14  [14,17] | 0.169 ^#^ |
|  |  |  |  |  |  |  |  |  |
| EtCO_2_  (mmHg) | 20 mEq/L | 94  [80,98] | 89  [82,95] | 90  [83,99] | 90  [80,102] | 90  [62,105] | 62  [53,75] | 0.195 * |
|  | 40 mEq/L | 84  [70,108] | 88  [69,124] | 93  [70,124] | 94  [69,126] | 95  [70,121] | 86  [51,131] | 0.611 ^#^ |
|  |  |  |  |  |  |  |  |  |
| PaO_2_  (mmHg) | 20 mEq/L | 104 [74,128] | 104 [81,123] | 115 [73,123] | 114 [89,129] | 115 [84,133] | 132 [121,146] | 0.308 * |
|  | 40 mEq/L | 117 [96,131] | 106 [83,132] | 128 [108,131] | 108 [96,129] | 127 [110,132] | 122 [81,144] | 0.368 ^#^ |

Data are shown as median [Percentile 25^th^,Percentile 75^th^].

§ Only the timepoints baseline, 1^st^, 2^nd^, and 3^rd^ hour were statistically analyzed.

* Mixed model timepoint vs. variable interaction.

# Mixed model group vs. variable interaction.
