# Supplementary material for: Effect of continuous dialysis on blood pH in acidemic hypercapnic animals with severe acute kidney injury: a randomized experimental study comparing high vs. low bicarbonate affluent
Source: Intensive Care Med Exp. 2017 May 30;5:28. doi: 10.1186/s40635-017-0141-6 (PMC5449359; doi:10.1186/s40635-017-0141-6)
Supplement: Supplementary file 6 — Hemodialysis quality assessment. Data are shown as the median [25th percentile, 75th percentile]. *Mixed model timepoint vs. variable interaction. #Mixed model group vs. variable interaction. (DOCX 21 kb) [file 40635_2017_141_MOESM6_ESM.docx]

| **Table S5: Hemodialysis quality assessment** | | | | | | |
| --- | --- | --- | --- | --- | --- | --- |
|  |  |  |  |  |  |  |
|  | Group | Baseline ^§^ | 1 hour ^§^ | 3 hours ^§^ | Last Hour | p value |
|  |  |  |  |  |  |  |
| BUN (mg/dL) | 20 mEq/L | 10  [7,15] | 9  [6,12] | 9  [6,11] | 9  [6,11] | 0.570* |
|  | 40 mEq/L | 10  [6,19] | 6  [4,14] | 6  [5,12] | 9  [6,15] | 0.597^#^ |
|  |  |  |  |  |  |  |
| Creatinine (mg/dL) | 20 mEq/L | 1.95  [1.58,2.55] | 2.00  [1.10,2.48] | 2.00  [1.40,2.33] | 1.9  [1.43,2.35] | 0.966* |
|  | 40 mEq/L | 1.60  [1.40,2.38] | 1.60  [1.40,1.75] | 1.60  [1.48,1.65] | 1.90  [1.50,2.50] | 0.587^#^ |
|  |  |  |  |  |  |  |
| FUN/BUN | 20 mEq/L | - | 0.84  [0.52,0.99] | 0.98 [0.57,1] | 0.84  [0.41,1.17] | 0.819* |
|  | 40 mEq/L | - | 0.89  [0.72,1] | 1  [0.97,1] | 0.79  [0.67,0.91] | 0.570# |
|  |  |  |  |  |  |  |
| Creatinine Clearance (ml/min) | 20 mEq/L | - | 24 [21,32] | 28 [12,34] | 26 [20,31] | 0.443* |
|  | 40 mEq/L | - | 26 [16,30] | 24 [20,27] | 27 [19,36] | 0.316^#^ |
|  |  |  |  |  |  |  |
| Pressure decay after filter (mmHg) | 20 mEq/L | - | 80 [62,97] | 79 [68,89] | 97 [75,107] | 0.856* |
|  | 40 mEq/L | - | 88 [74,108] | 89 [70,111] | 80 [84,110] | 0.534^#^ |
|  |  |  |  |  |  |  |
| Blood Flow (ml/min) | 20 mEq/L | - | 194  [177,200] | 193 [176,200] | 195 [175,207] | 0.679* |
|  | 40 mEq/L | - | 182 [150,214] | 178  [162,215] | 176 [153,218] | 0.916# |
|  |  |  |  |  |  |  |
| Cumulative Effluent (ml) | 20 mEq/L | - | 1750 [1525,2275] | 5350 [4743,5900] | 14895 [8400,16975] | 0.040* |
|  | 40 mEq/L | - | 1700 [1400,2000] | 5200 [4100,6100] | 17100 [14700,20450] | 0.544^#^ |
|  |  |  |  |  |  |  |
| Cumulative Affluent (ml) | 20 mEq/L | - | 1550 [1200,2000] | 4950 [4575,5300] | 14600 [9225,16400] | <0.001* |
|  | 40 mEq/L | - | 1600 [1200,1800] | 4800 [4350,5350] | 16400 [14400,20550] | 0.754^#^ |

Data are shown as median [Percentile 25^th^,Percentile 75^th^].

* Mixed model timepoint vs. variable interaction.

# Mixed model group vs. variable interaction.
